# An Effective Method to Identify Shared Pathways and Common Factors among Neurodegenerative Diseases

**DOI:** 10.1371/journal.pone.0143045

**Published:** 2015-11-17

**Authors:** Ping Li, Yaling Nie, Jingkai Yu

**Affiliations:** 1 National Key Laboratory of Biochemical Engineering, Institute of Process Engineering, Chinese Academy of Sciences, Beijing, 100190, China; 2 University of Chinese Academy of Sciences, Beijing, 100049, China; National Institutes of Health, UNITED STATES

## Abstract

Groups of distinct but related diseases often share common symptoms, which suggest likely overlaps in underlying pathogenic mechanisms. Identifying the shared pathways and common factors among those disorders can be expected to deepen our understanding for them and help designing new treatment strategies effected on those diseases. Neurodegeneration diseases, including Alzheimer's disease (AD), Parkinson's disease (PD) and Huntington's disease (HD), were taken as a case study in this research. Reported susceptibility genes for AD, PD and HD were collected and human protein-protein interaction network (hPPIN) was used to identify biological pathways related to neurodegeneration. 81 KEGG pathways were found to be correlated with neurodegenerative disorders. 36 out of the 81 are human disease pathways, and the remaining ones are involved in miscellaneous human functional pathways. Cancers and infectious diseases are two major subclasses within the disease group. Apoptosis is one of the most significant functional pathways. Most of those pathways found here are actually consistent with prior knowledge of neurodegenerative diseases except two cell communication pathways: adherens and tight junctions. Gene expression analysis showed a high probability that the two pathways were related to neurodegenerative diseases. A combination of common susceptibility genes and hPPIN is an effective method to study shared pathways involved in a group of closely related disorders. Common modules, which might play a bridging role in linking neurodegenerative disorders and the enriched pathways, were identified by clustering analysis. The identified shared pathways and common modules can be expected to yield clues for effective target discovery efforts on neurodegeneration.

## Introduction

Healthcare improvements coupled with low fertility are expected to cause an increasingly larger proportion of old population, which leads to more chronic illnesses [1]. A representative type of chronic disease is neurodegenerative disorders, such as Alzheimer’s disease (AD), Parkinson’s disease (PD) and Huntington's disease (HD). Neurodegenerative diseases bring enormous suffering in terms of economical cost and emotional trauma. Unfortunately, the etiologies and pathogeneses of these disorders remain not well understood. Current therapies for these diseases are palliative rather than curative and their effectiveness is still far from satisfactory [2]. It is thus critical to elucidate factors underlying these disorders for better design of intervention strategies. However, the traditional strategy of “one disease-one target-one drug” is no longer effective and challenged in many cases, especially with regard to multi-factorial diseases [3, 4], which is the case for neurodegenerative disorders. Physiological redundancies in biological networks could also limit efficacy of administered drugs [5]. For complex diseases, multiple targets or pathways have to be affected for successful treatment outcomes.

AD, PD and HD share at least two common symptoms: motor and cognitive impairment [6–8]. Similar phenotypic traits suggest that there are likely overlaps in the pathogenic mechanisms underlying distinct neurodegenerative disorders. Compared to studying individual diseases separately, identification and analysis of the common dysfunctional proteins or dysregulated modules/pathways of the three diseases can be expected to provide deeper insights into their pathogenic processes. Understanding the common pathogenic processes could facilitate efforts to design treatment strategies utilizing optimal drug combinations that could work effectively for the diseases.

**Fig 1:**
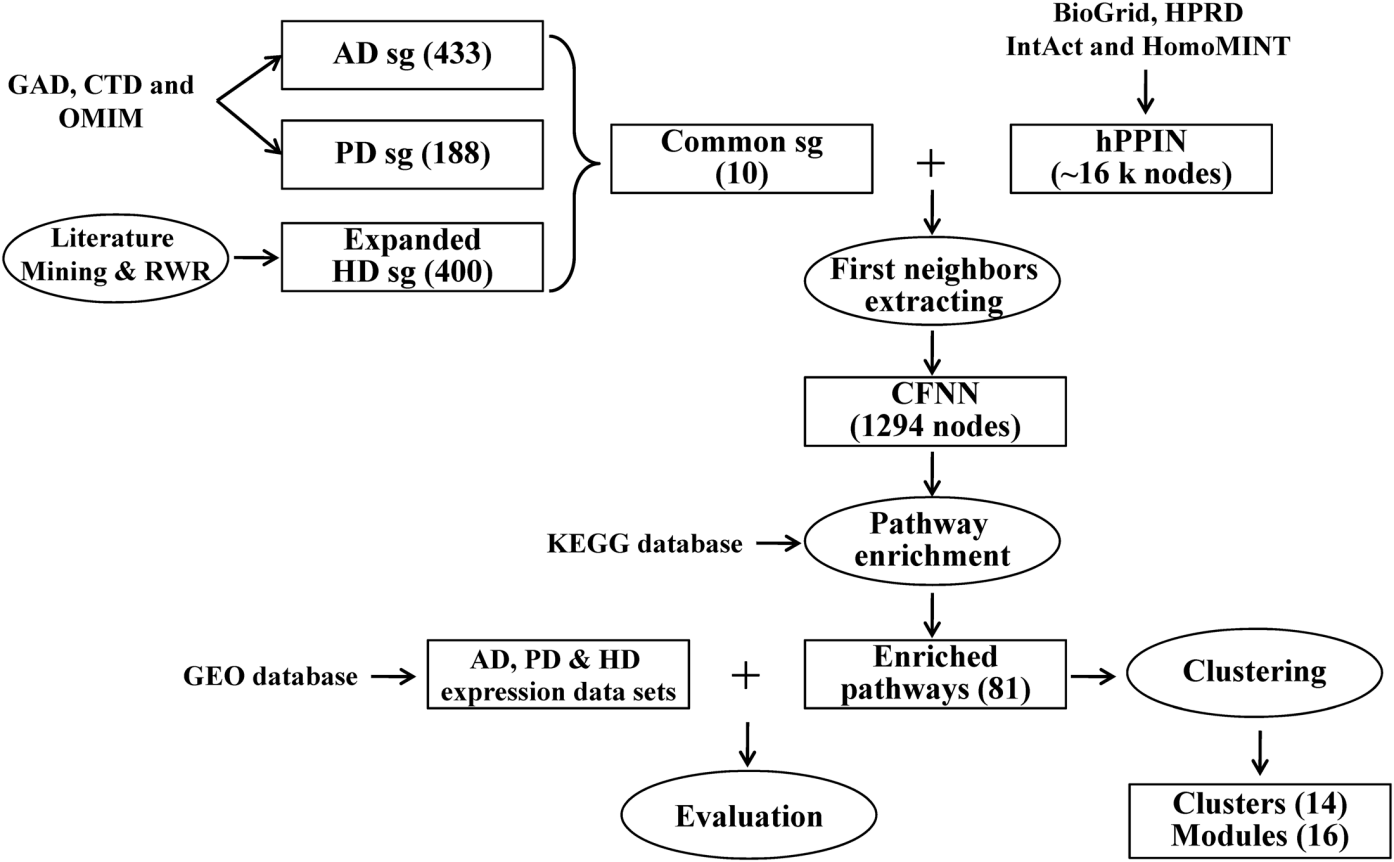
Workflow for identification of shared pathways and common modules among AD, PD and HD. In the first step, AD, PD and HD susceptibility gene (sg) sets were collected and their intersection were defined as common sg. Meanwhile, common sg's first neighbors in the human protein-protein interaction network (hPPIN) was extracted to construct common gene first neighbor network (CFNN). Then, KEGG pathway enrichment analysis was applied to the nodes in CFNN to get shared pathways of AD, PD and HD, following by gene expression analysis to evaluate the found pathways. Finally, hierarchal clustering was applied to cluster the enriched pathways and indentify common modules in CFNN. RWR: random walk with restart.

Differentially expression genes (DEG) and genome-wide association studies (GWAS) are usually applied to study related biological pathways of a specific disease. For multiple diseases, however, there is lack of effected method to study their shared pathways and common factors. In this paper, we proposed a simple and effective approach which integrated common susceptibility genes of multiple disorders and the human protein-protein interaction data (Fig 1). AD and PD susceptibility genes were acquired from public online databases. HD susceptibility genes were acquired through literature mining and the random walk algorithm [9]. Common genes of the three susceptibility gene sets and their first neighbors in the human protein-protein interaction network (hPPIN), called as CFNN, were extracted to perform pathway enrichment analysis, which identified pathways related with neurodegenerative diseases. Gene expression data sets from NCBI GEO database [10] were applied to evaluate the computed pathways. Meanwhile, pathway clustering analysis obtained the common modules in CFNN shared by distinct pathways. Those modules might play a bridging role in linking enriched pathways and neurodegeneration.

## Materials and Methods

### Data source

Human protein-protein interaction network (hPPIN) was constructed by integrating four existing databases, i.e., BioGrid [11], HPRD [12], IntAct [13], and HomoMINT [14]. Protein identifiers were mapped to the genes coding for the proteins, and redundant interactions were removed. The comprehensive protein-protein interaction network covers 15,710 human genes and 143,237 interactions.

AD and PD susceptibility genes were acquired from the GAD [15], CTD [16] and OMIM [17] database. These public data sources store associations between genes and diseases, but focus on different aspects of the phenotype-genotype relationship. After integrating all the records in the databases, 433 and 188 distinct susceptibility genes were collected for AD and PD, respectively. The three databases does not have sufficient data for HD, whose susceptibility genes were collected by text-mining of biomedical literatures from PubMed (http://www.ncbi.nlm.nih.gov/pubmed/). It produced 20 HD susceptibility genes. Compared with AD and PD, the number of collected HD susceptibility genes is still rather low, which might be due to the much lower prevalence of HD than AD and PD [18–20]. To bring the number of HD's susceptibility genes to the same level as those of AD and PD, a random walk algorithm [9] was applied to expand the number of HD susceptibility genes through the hPPIN, using manually collected HD susceptibility genes as seed nodes. The top 400 genes ranked by random walk (including the seed genes) were selected as the expanded set of HD susceptibility genes.

### Random walk with restart (RWR)

RWR is a variant of random walk. It mimics an iterative walker that moves from a current node to a randomly selected adjacent node, and allows the restart of the walk in every time step at source nodes with predefined probability *γ* [9]. RWR is formally defined as follows:

$$p_{t+1}=(1-\gamma )Wp_{t}+\gamma p_{0}$$

Where *W* is the column-normalized adjacency matrix of the graph and *p*_*t*_ is a vector in which the *i*th element holds the probability of being at node *i* at time step *t*. *p*_*0*_ is the initial probability vector where equal probabilities were assign to the source nodes, with the sum of the probabilities equal to 1.

In this study, RWR was used to prioritize susceptibility genes from among genes that have not been associated with HD. The set of source nodes consists of genes known to be associated with HD. The predefined probability *γ* was set to 0.75, as was done by Kohler et al [21]. All genes in the network are eventually ranked according to their steady-state probabilities and the top 400 genes were selected.

### Common susceptibility genes and their first neighbor network construction

We took the intersection of AD, PD and expanded HD susceptibility genes and called it the set of common susceptibility genes of the three disorders. To check the significance of those common genes, we randomly generated three gene sets of the same size as that of AD, PD and expanded HD susceptibility genes from hPPIN and computed the number of common genes among them. The process was repeated 10^4^ times. A p-value was then computed for the observed number of common genes.

Nearest neighbors of the common genes were extracted from the hPPIN to construct a network consisting of the common genes and their first neighbors, which was called the Common gene First Neighbor Network (CFNN).

### Pathway enrichment and clustering analysis

CFNN consists of the common susceptibility genes and their direct interaction partner in hPPIN. Pathways enriched with genes in CFNN are very likely shared pathways of AD, PD and HD. ClueGO v2.0.7 [22] was used to perform KEGG [23] pathway enrichment for all nodes in CFNN. ClueGO, an Cytoscape [24] plug-in, can identify biological pathways enriched with a list of genes. Two-sided (enrichment/depletion) method based on the hypergeometric distribution was used for statistical test with a multiple testing p-value correction using the Benjamini-Hochberg method [25]. Pathways with adjusted p-value < 0.05 were regarded as related biological pathways to CFNN genes and were selected for further analysis.

**Fig 2:**
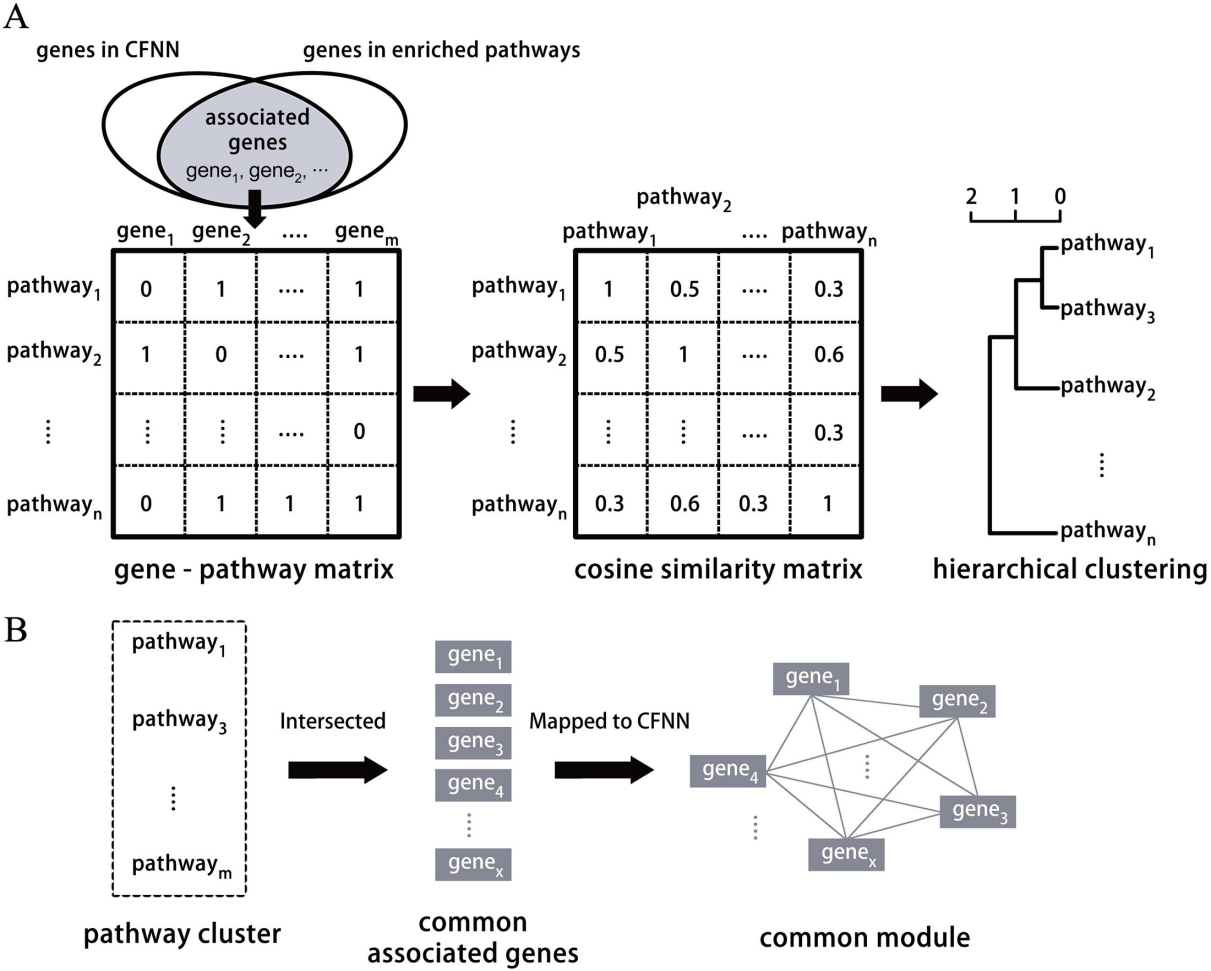
Pathway clustering analysis and common module extracting. (A) Diagram of clustering analysis. Hierarchical clustering was applied and only associated genes in a specific pathways were considered, not the full genes. (B) Extracting common module from a pathway cluster. The common module is a part of CFNN, and might playing a bridging role in linking enriched pathways and neurodegeneration.

Hierarchical clustering approach was use for clustering analysis. Genes appearing in both the CFNN and enriched KEGG pathways were named as **associated genes** (Fig 2(A)). A binary associated gene-pathway matrix was created (0: absent, 1: present). Based on this matrix, a cosine similarity matrix of pathways was built and used to group the pathways into clusters. To getting meaningful clusters, we manually checked the dendrogram plot of results and chose clustering distance *d* = 1.1 as the final cutting point. For each cluster, each member pathway's associated genes were intersected to obtain their common associated genes. Those common associated genes were then mapped to CFNN to get their interaction subnetwork, called common module (Fig 2(B)). The average clustering coefficients of the acquired modules were computed.

### Gene expression analysis

Twenty AD, PD and HD gene expression data sets (March 16, 2014), attached raw data, were collected from the NCBI GEO database (see S1 File). Among those extracted expression sets, only GSE7621 [26], GSE8397 [27], GSE20168 and GSE20292 [28, 29] on PD patients and GSE45596 [30] on AD patients (see Table 1), have significantly differentially expressed genes (methods were explained below). 4 of the 5 expression sets were on PD *vs*. Normal. The combine of differentially expressed genes acquired in the 4 expression sets were defined as the finally differentially expressed gene set on PD.

**Table 1:** List of selected gene expression data sets.

GEO accession	Sample tissue	Platform	Nr. of Sig. Diff. ^a^
PD *vs*. Normal			
GSE7621	Substantia nigra	HG-U133_Plus_2	143
GSE8397	Substantia nigra	HG-U133A/B	655
GSE20168	Prefrontal area	HG-U133A	169
GSE20292	Substantia nigra	HG-U133A	24
AD *vs*. Normal			
GSE45596	Brain microvessel	Agilent-014850	2063

^a^: The number of significantly differentially expressed genes.

For Affymetrix HG_U133 (including A chip and B chip) and HG-U133_Plus_2 platform, the CEL source files were preprocessed by the RMA algorithm with default parameters in the R Bioconductor package[31]. For Agilent-014850 platform, preprocessing steps of the TXT source files included background correction with the “normexp” method to subtract the background intensity from the foreground intensity for each spot [32], within-array normalization with the “loess” method to normalize the M-values for each array separately, and between-array normalization with the “quantile” method to normalize intensities or log-ratios for them to be comparable across arrays [33]. The package limma [34] in Bioconductor was then used to perform differential expression analysis for the preprocessed microarray data. Probe sets were mapped to NCBI entrez genes using R package GEOquery [35]. In cases where there were multiple probe sets that correspond to the same gene, expression values of those probe sets were averaged. Genes that were significantly differentially expressed with a Benjamini and Hochberg adjusted p-value less than 0.05 [25] were picked for later analysis.

To evaluate the enriched KEGG pathways, each node of the pathway was considered as a component. Those components were a mixture of one protein node and multi-protein node. Multi-protein component, which contains more than one protein, was also regarded as a single component. That is to say, if any individual protein of the multi-protein component was found to be significantly differentially expressed in gene expression analysis, the corresponding multi-protein component was taken as significantly differentially expressed. For example, α-Catenin, a multi-protein component in adherens junction, is composed of catenin alpha-1, catenin alpha-2 and catenin alpha-3. If one of the three proteins was shown to be significantly differentially expressed, α-Catenin was defined as a significantly differentially expressed component. Gene symbols of proteins involved in all components were extracted from KEGG. To check the significance of obtaining those differentially expressed components in an enriched pathway, we randomly generated gene set of the same size as that of computed differentially expressed genes from human gene set, and computed the number of components involved in the enriched pathways. The process was repeated 10^4^ times. A p-value was then computed for the observed number of differentially expressed components.

## Results and Discussion

### Common susceptibility genes of AD, PD and HD show high statistical significance

**Fig 3:**
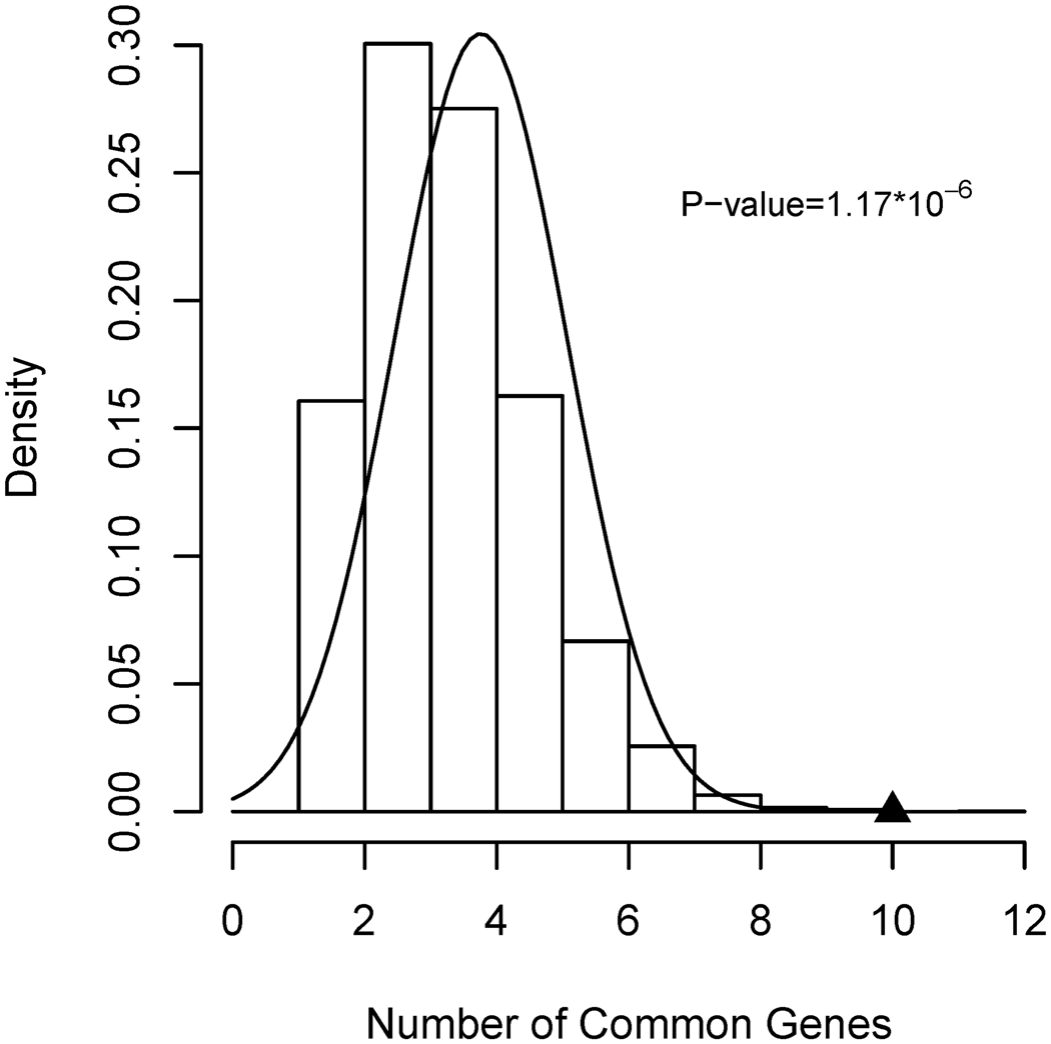
Probability density for obtaining common genes. The observed value is marked with a filled triangle.

AD, PD and HD share 10 common susceptibility genes, which were obtained by taking intersection of susceptibility gene sets of the three disorders. P-value for finding same or larger size of common gene set was found to be 1.17×10^−6^ (Fig 3), showing that the acquired 10 common genes was statistically significant.

Table 2 showed clinical indications for 5 of the 10 common genes. Interestingly, three of them had been used to treat cancers, i.e., PARP1, GSK3B and UCHL1. It suggests that cancers and neurodegenerative disorders could be correlated. GSK3B, UCHL1 and LRRK2 were also reported to be potential therapeutic targets for neurodegenerative diseases and inhibitors had been designed against them [36–38]. The remaining 5 common genes showing no indication yet were all related with key processes in neurodegeneration. CASP3, FAS, SQSTM1 and YWHAZ participate in cell apoptosis [39, 40], which are activated in neurodegenerative diseases [41]. TFAM, playing a role in organizing and compacting mitochondrial DNA, is related with the mitochondrial dysfunction in neurodegenerative disorders [42]. The 10 common genes acquired here might be a good starting point to find overlapped pathogenic mechanisms underlying the three diseases, facilitating efforts to discover potential drug targets for neurodegenerative diseases.

**Table 2:** Indications of common susceptibility genes of AD, PD and HD.

Gene symbol	Protein	Indication^a^
ESR2	Estrogen receptor beta	**Successful target:** Vasomotor symptoms
PARP1	Poly [ADP-ribose] polymerase-1	**Successful target:** Inflammatory skin conditions; **Clinical trial target:** Malignant melanoma, Triple negative breast cancer, Non small cell lung cancer, Brain cancer, Stroke, Myocardial infarction
GSK3B	Glycogen synthase kinase-3 beta	**Clinical trial target:** Non Hodgkin lymphoma, Glioblastoma multiforme, Acute promyelocytic leukemia, Brain and central nervous system tumors; **Research target**: AD, Type II diabetes
UCHL1	Ubiquitin carboxyl-terminal hydrolase isozyme L1	**Research target:** Cancer, AD and PD
LRRK2	Leucine-rich repeat serine/threonine-protein kinase 2	**Research target:** PD

^a^: Source from Therapeutic Target Database [43].

### Eighty-one KEGG pathways were enriched with common susceptibility genes and their nearest neighbors in hPPIN

The CFNN covers 1294 human genes with 21679 interactions. 81 KEGG pathways were enriched with adjusted p-value < 0.05. 574 genes were found to be associated with CFNN and enriched KEGG pathways, called the **associated genes** (see Fig 2). The list of enriched KEGG pathways and their associated genes can be found in S2 File.

**Fig 4:**
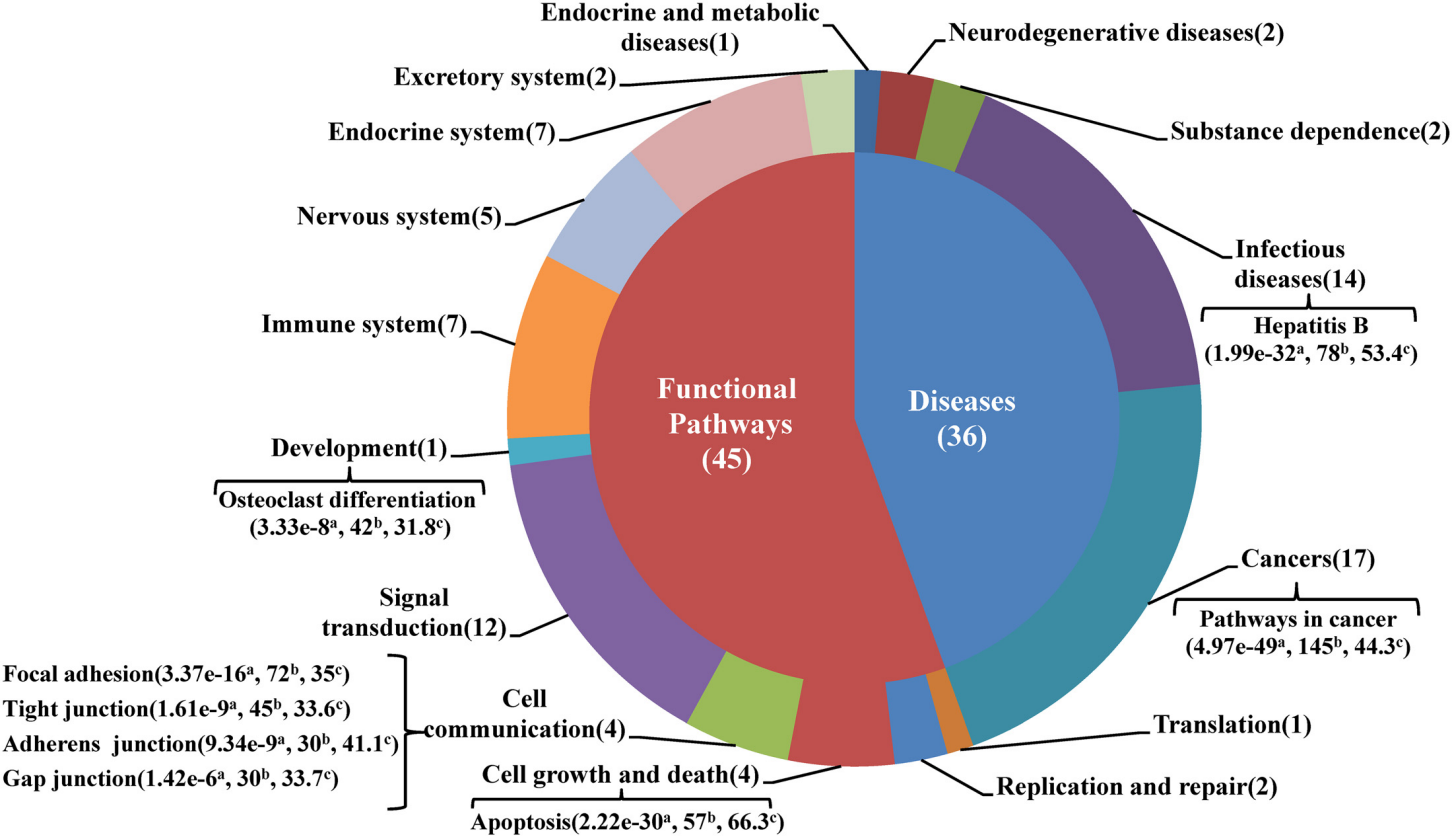
Subclasses of enriched KEGG pathways. The number of pathways belong to each subclass is shown in parentheses. Pathways mentioned in section 3.2 are labeled. ^a^: Benjamini and Hochberg adjusted p-value; ^b^ and ^c^: Number and percentage of associated genes in each pathway. Percentage represents the proportion of associated genes in all known genes involved in a pathway.

The enriched pathways belonged to two categories: functional pathways and diseases (Fig 4). Thirty six were human disease pathways, which belonged to 5 types of diseases: cancers, infectious diseases, neurodegenerative diseases, endocrine and metabolic diseases, and substance dependence. Among those, cancers and infectious diseases were the two largest subclasses, which had 17 and 14 disease pathways respectively (Fig 4). The two most significantly enriched human disease pathways were pathways in cancer and hepatitis B, with adjusted p-values of 4.97×10^−49^ and 1.99×10^−32^ respectively (Fig 4). Pathways in cancer is a KEGG overview pathway which integrates all specific KEGG cancer pathways' signaling networks. It is actually not surprising to see many cancers and infectious diseases related to neurodegeneration. Although neurodegenerative disease and cancer are two distinct pathological disorders, past epidemiological studies suggest that sufferers of neurodegenerative disorders have reduced incidence for most cancers [44–46]. Moreover, a growing body of evidence shows that these two types of diseases share common mechanisms of genetic and molecular abnormalities, which involve regulation of cell cycle, DNA repair, protein turnover, oxidative stress, and autophagy [47]. Many studies have also shown that viral and bacterial infections can induce significant neuronal dysfunction and degeneration of specific neuronal populations [48]. It was reported that viruses could induce brain dysfunction by either direct cytolytic effects or bystander inflammatory reactions, especially by neurotropic viruses (for example, measles, herpesviridae and influenza) [49]. Recently, Deleidiet al. raised a hypothesis that viral infections and inflammation prime neurons and immune cells in the brain, rendering neuronal populations vulnerable to degeneration in the face of subsequent insults [50].

The remaining 45 were miscellaneous functional pathways, which could be divided into 10 subclasses: signal transduction, immune system, endocrine system, nervous system, cell communication, cell growth and death, excretory system, replication and repair, translation, and development (Fig 4). Pathway apoptosis was found with an very high p-value of 2.22×10^−30^ (Fig 4). It is known that neuronal death underlies the symptoms of many neurodegenerative disorders including Alzheimer’s, Parkinson’s and Huntington’s diseases. Early research had shown that apoptosis, involving oxidative stress, perturbed calcium homeostasis, mitochondrial dysfunction and activation of cysteine proteases called caspases, is a shared pathway of AD, PD and HD [51]. The newly discovered immune channel of brain [52] suggests possible critical role of immune system in etiology of neurodegenerative disorders. In fact, immune system was found to be a main subclass of functional pathways enriched with genes of neurodegeneration diseases (Fig 4). Immune system’s role in the initiation of neuronal degeneration has been documented for HD, and activation of microglia (brain macrophages) is associated with cognitive dysfunction [53, 54]. Immune activation has also been indicated in the early phases of AD [55]. Moreover, several studies in rodent models of PD demonstrated that neuroinflammation can precipitate PD-like pathology [56–61].

Interestingly, correlation was also found between osteoclast differentiation and neurodegenerative disorders. Osteoclast differentiation was the only pathway in development subclass that was enriched. There were 42 associated genes (nearly one-third of osteoclast differentiation genes) and the adjusted p-value was 3.33×10^−8^ (Fig 4). The osteoclasts, multinuclear cells originating from the hematopoietic monocyte-macrophage lineage, are responsible for bone resorption. Epidemiological studies showed that patients with AD had an increased risk of developing osteoporotic hip fractures [62]. Quite recently, it was found that amyloid beta peptide in patients with AD was elevated in osteoporotic bone tissues and enhances osteoclast function [63]. Our findings, combined with previously published results, suggest that osteoclast differentiation pathway may be a common factor for both osteoporosis and neurodegeneration.

Focal adhesion and gap junction, members of the cell communication group, had been reported to be related to neurodegenerative diseases [64–66]. In the case of the remaining two pathways in the cell communication group (Fig 4), i.e., adherens junction and tight junction, little research was found on their relationship with neurodegeneration. Our results, however, showed adherens junction and tight junction also had significant correlation with neurodegenerative disorders. The number of associated genes of adherens junction and tight junction were 30 and 45, with p-values of 9.34×10^−9^ and 1.61×10^−9^, respectively (Fig 4).

### Gene expression analysis confirmed that adherens and tight junctions were indeed correlated with neurodegeneration

After gene expression analysis, 927 significantly differentially expressed genes for PD and 2063 for AD were obtained. The list of differentially expressed genes can be found in S3 File.

**Fig 5:**
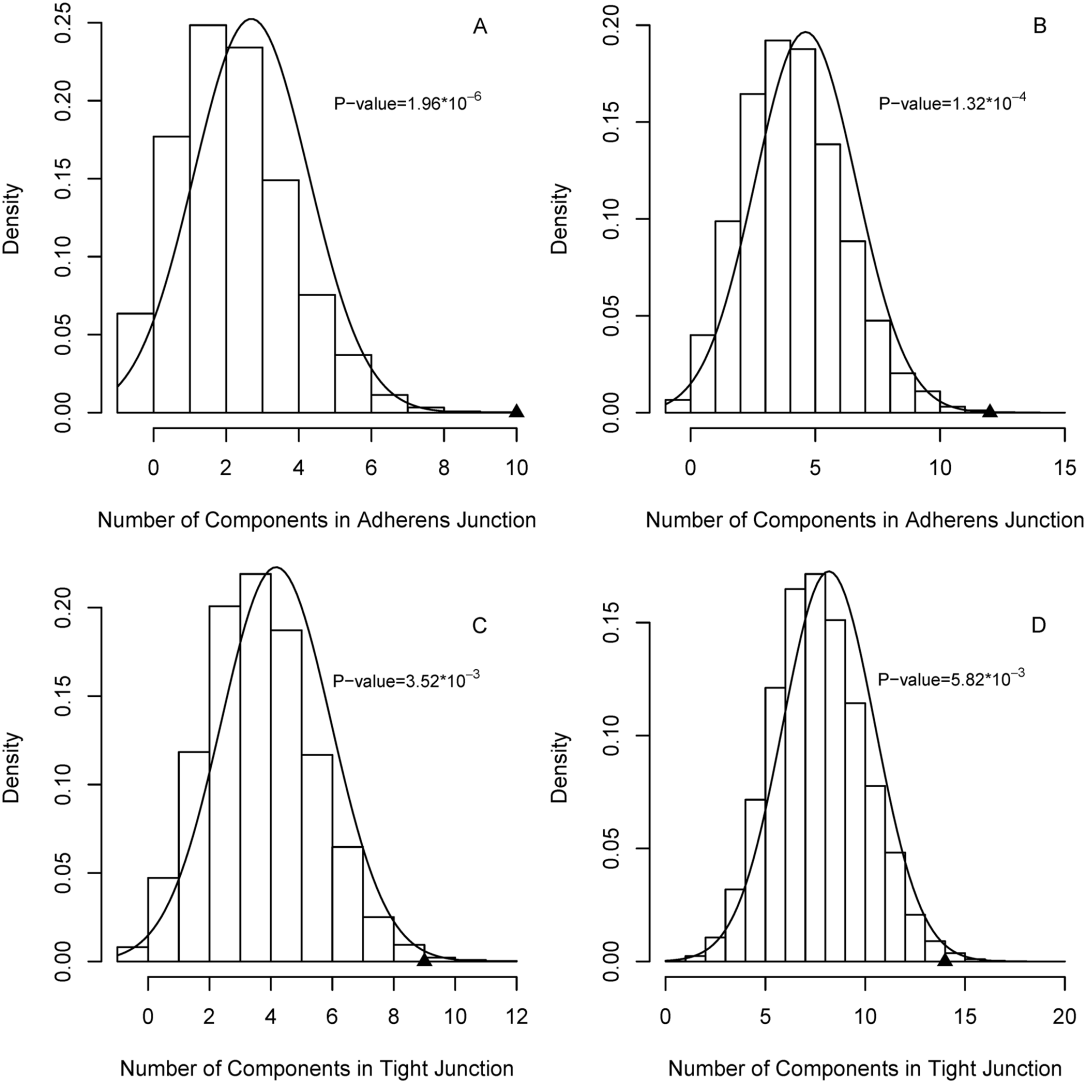
Probability density for obtaining the number of components in adherens and tight junction pathways. (A) and (C) for PD, (B) and (D) for AD; the observed value is marked with a filled triangle.

Each of adherens and tight junctions had 50 pathway components (see S4 File for details and section 2.5 for the definition of "component"). For the PD differentially expressed gene set, adherens and tight junction had 10 and 9 differentially expressed components, respectively. For AD, the numbers of differentially expressed components were 12 and 14. For adherens junction, p-values for obtaining the number of components in PD and AD were 1.96×10^−6^ and 1.32×10^−4^ (Fig 5(A) and 5(B)). For tight junction, the p-values were 3.52×10^−3^ and 5.82×10^−3^ (Fig 5(C) and 5(D)). The small p-values imply that the number of differentially expressed components is statistically significant for the two junction pathways. Pathway enrichment (section 3.2) and gene expression analysis together indicated that adherens and tight junction are very likely related to neurodegenerative diseases. Actually, adherens and tight junction were found to be involved in maintaining blood-brain barrier (BBB) integrity [67]. It had been shown that changes in BBB existed in AD and PD patients [68]. The two junction pathways may deserve more attention for better understanding of neurodegenerative processes.

### Common modules behind the enriched pathways were identified through clustering analysis

**Fig 6:**
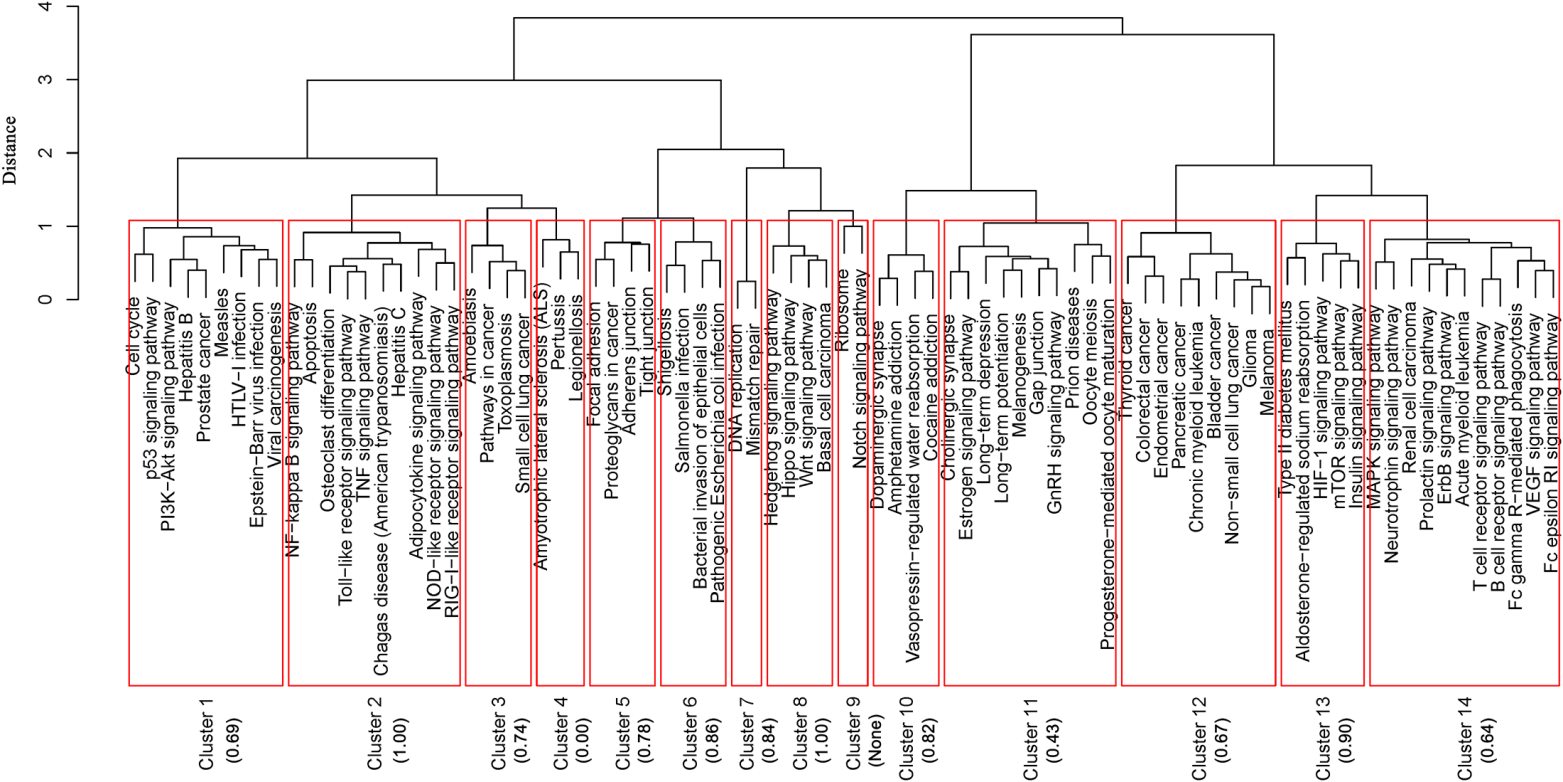
Hierarchical clustering of the enriched pathways. Each cluster is encircled by a rectangle and numbered. Clustering coefficient of common module in each cluster is shown in parentheses. None: represent the fact that there are no common associated genes within the corresponding cluster.

Section 3.2 had shown that the enriched pathways were interconnected, such as infectious diseases and immune system. Seeking out the molecular connections among those pathways might help to illustrate their relationship with neurodegenerative diseases, lead to deeper sight into the pathogenic process of neurodegeneration, which could then facilitate designing of effective synergistic treatment strategies. Clustering analysis was utilized to explore internal connections of the enriched pathways. Fig 6 shows result of the hierarchical clustering based on the cosine similarity of associated gene vectors. 14 clusters were finally acquired, which showed significant differences from the KEGG categories. Some clusters were composed of functional pathways and diseases, e.g., cluster 1 and cluster 2. For others, pathways belonged to different subclasses were clustered together, e.g., cluster 3, cluster 4 and cluster 10. The common associated genes within each cluster and their interaction network, called as common module, were extracted. The extracted common module was also a part of CFNN, because the associated genes were obtained by taking intersection of CFNN and the enriched pathways (Fig 2). Those modules were connected denser than CFNN. The mean clustering coefficient of them was 0.65 (Fig 6), while clustering coefficient of CFNN was only 0.38. The found modules could thus be the local cores within CFNN and might play a bridging role between pathways in a cluster and neurodegeneration. Elucidating working mechanisms of the modules, how they control those related pathways, may provide a fruitful strategy for understanding neurodegenerative disorders.

**Fig 7:**
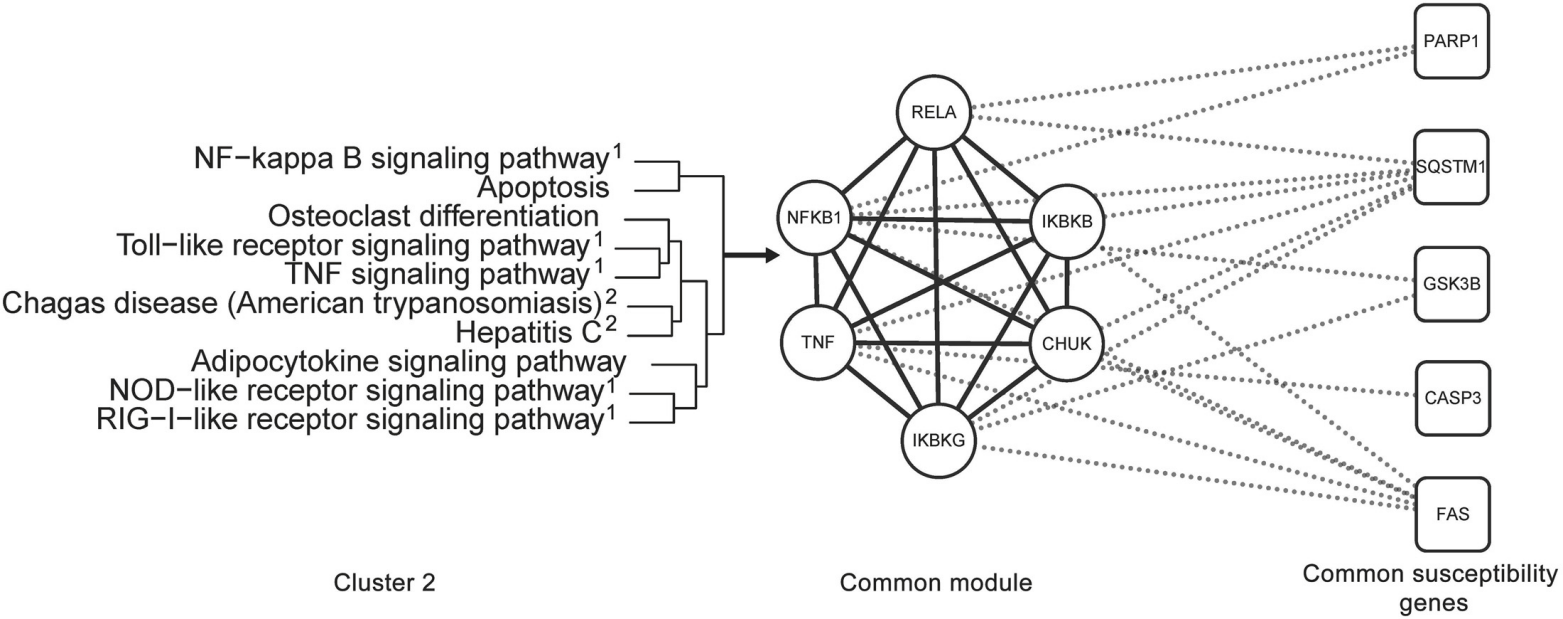
The common module from cluster 2. Their relationship with common susceptibility genes of AD, PD and HD is depicted. Module inner interactions are labeled by solid line, its relationship with susceptibility genes by dashed line. Gene names are marked on the nodes. ^1^: pathways related to inflammation, ^2^: pathways involved in infectious diseases.

As an example, Fig 7 showed the acquired common module from cluster 2. The module's relationship with common susceptibility genes of AD, PD and HD was also shown. The common module, which happens to be a fully connected network, was composed of RELA, NFKB1, IKBKB, TNF, CHUK and IKBKG. Half of the pathways in cluster 2 (Fig 7) had been found to be directly related to inflammation. Chagas disease and Hepatitis C were involved in infectious diseases. Inflammation and infectious diseases had been shown to be correlated with neurodegeneration. Our study also showed that Osteoclast differentiation might be a common pathway for both osteoporosis and neurodegeneration (section 3.2). The extracted common module's dysfunction, caused by dysregulation of common susceptibility genes, may be a key contributing factor for neurodegenerative disorders, inflammation, infectious diseases and osteoporosis. The found module role in neurodegeneration could thus deserve more in-depth research. Detailed information about other common modules can be found in S1 Fig.

## Conclusion

The traditional drug discovery paradigm of attempting to design precise drugs hitting single targets has seen itself challenged for treatment of complex diseases. The less than perfect efficacy of the single target, single drug approach is mainly due to drug promiscuity, off-target effects, and biological pathway redundancy/robustness. Apparent similarities among groups of closely related disorders hint at possible overlaps in their underlying mechanisms. Figuring out common factors and network modules shared within a group of distinct but related diseases may allow us to pinpoint the fundamental factors responsible for the group of disorders. Computed relationship among pathways of related diseases can assist understanding of their etiology; correlations between the shared pathways with other biological processes/disorders can facilitate drug discovery efforts by suggesting possible treatment candidates for drugs already approved (drug repositioning).

Neurodegenerative disorders including AD, PD and HD were taken as a case study. Their susceptibility genes were collected to compute biological pathways related with neurodegeneration. 81 KEGG pathways were found to be enriched with neurodegenerative genes. Those pathways were involved in cancers, infectious diseases, apoptosis, osteoclast differentiation, and immune system. Sufficient evidences exist for the found correlation between neurodegeneration and the aforementioned pathways. Our work also showed that adherens and tight junctions, part of the cell communication process, were also correlated with neurodegeneration. Gene expression analysis confirmed that the two junction pathways were indeed correlated with neurodegeneration. The approach applied in this paper can thus be expected to find non-obvious pathways related with a group of closely related disorders. All of these show that a combination of common susceptibility genes and hPPIN is an effective method to study shared pathways involved in a group of related diseases. Not only the functional pathways related with them, but their relationships with other diseases. Moreover, the computed shared pathways can provide mechanistic hypotheses which can guide confirmatory testing to deepen our understanding of the diseases. Common modules bridging distinct pathways were identified by clustering analysis. Those bridging modules may be key points in linking together neurodegeneration and other pathways. Detailed study of the modules may provide potential targets to treat groups of related disorders simultaneously.

## Supporting Information

S1 File List of collected gene expression data sets of AD, PD and HD (XLS)

S2 File Detailed information of enriched pathways, including the list of associated genes. (XLS)

S3 File List of significantly expressed genes in AD and PD. (XLS)

S4 File The components of adherens and tight junction. (XLS)

S1 Fig The common modules in each cluster. (TIFF)
