# Supplementary figures and images for: An Effective Method to Identify Shared Pathways and Common Factors among Neurodegenerative Diseases
Source: PLoS One. 2015 Nov 17;10(11):e0143045. doi: 10.1371/journal.pone.0143045 (PMC4648499; doi:10.1371/journal.pone.0143045)

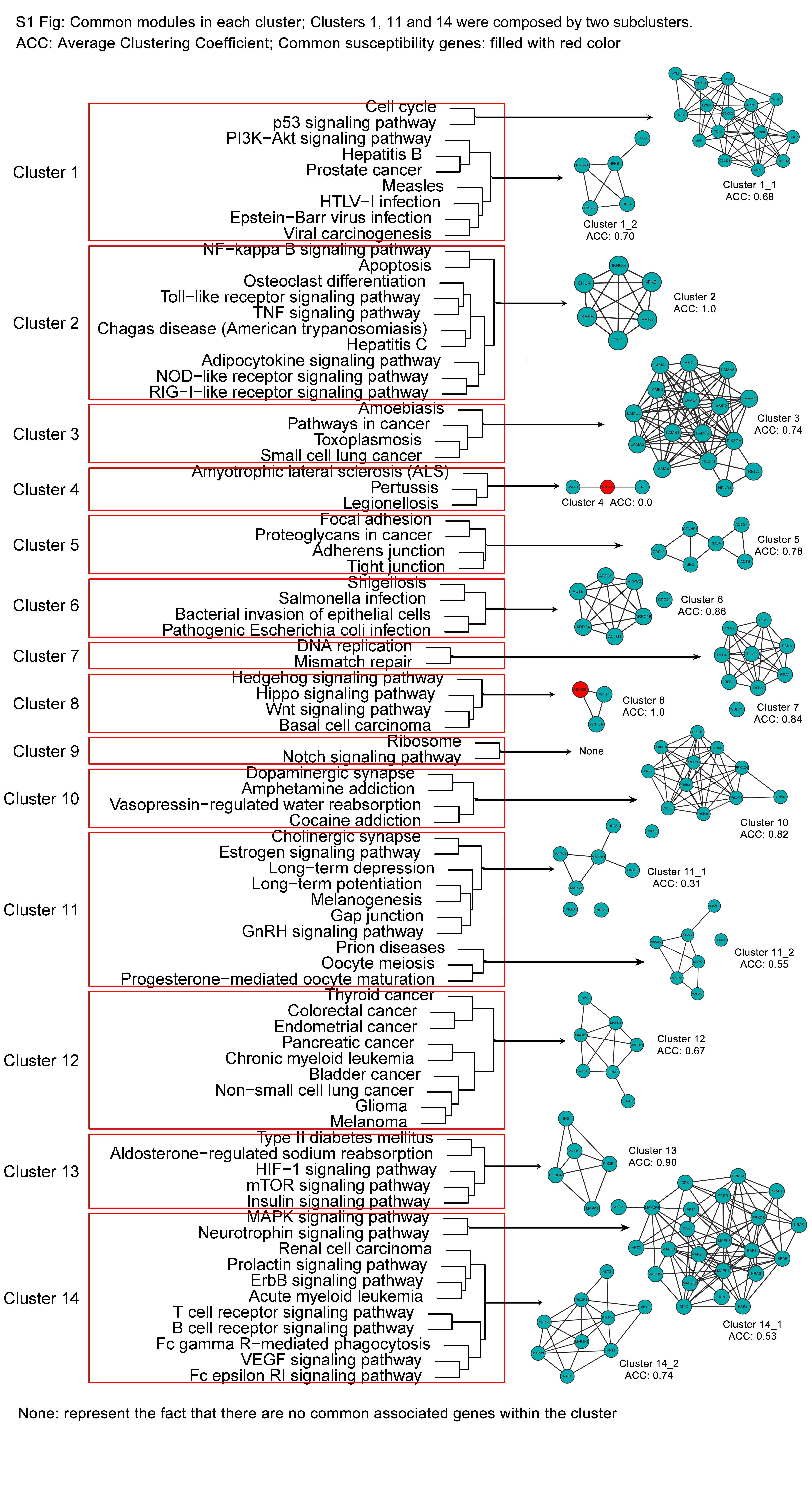

Supplement: S1 Fig — (TIFF) [file pone.0143045.s005.tiff]
